# Public Stigma against People with Mental Illness in the Gilgel Gibe Field Research Center (GGFRC) in Southwest Ethiopia

**DOI:** 10.1371/journal.pone.0082116

**Published:** 2013-12-04

**Authors:** Eshetu Girma, Markos Tesfaye, Guenter Froeschl, Anne Maria Möller-Leimkühler, Norbert Müller, Sandra Dehning

**Affiliations:** 1 Department of Health Education and Behavioral Sciences, Jimma University, Jimma, Ethiopia; 2 CIHLMU Center for International Health, Ludwig-Maximilians-Universität, Munich, Germany; 3 Department of Psychiatry, Jimma University, Jimma, Ethiopia; 4 Department of Infectious Diseases and Tropical Medicine, Ludwig-Maximilians-Universität, Munich, Germany; 5 Department of Psychiatry and Psychotherapy, Ludwig-Maximilians-Universität, Munich, Germany; Institute of Neuroepidemiology and Tropical Neurology, France

## Abstract

**Background:**

Public understanding about mental illnesses and attitudes towards people with mental illness (PWMI) play a paramount role in the prevention and treatment of mental illness and the rehabilitation of PWMI. The aim of this study was to measure public stigma against PWMI and the factors associated with stigma in the Gilgel Gibe Field Research Center (GGFRC) in Southwest Ethiopia.

**Methods:**

This community-based, cross-sectional study was conducted from June to August 2012 among 845 randomly selected respondents by using the Community Attitudes towards the Mentally Ill (CAMI) scale, an interviewer-administered questionnaire. Data was entered with EPI-DATA and then exported to STATA for analysis. Simple descriptive and linear regression analyses were performed to identify predictors of stigma against PWMI.

**Results:**

Of the total of 845 respondents, 68.17% were from rural districts. The mean stigma score was 2.62 on a 5-point score. The majority of the respondents (75.27%) believed that mental illness can be cured. Stress, poverty, and rumination were the most often perceived causes of mental illness. Rural residents had significantly higher stigma scores (std. β = 0.61, P<0.001). A statistically significant inverse relationship was found between the level of education and degree of stigma (std. β = −0.14, P<0.01), while higher income was significantly associated with more stigma (std. β = 0.07, P<0.05). Respondents with higher scores for perceived supernatural causes (std. β = −0.09, P<0.01) and perceived psychosocial and biological causes (std. β = −0.14, P<0.001) had significantly lower stigma levels.

**Conclusions:**

The study found a more undermining but less avoidant attitude towards PWMI. Rural residents showed higher levels of stigma. Stigma against PWMI was lower in people with an explanatory concept about the causes of mental illness and a higher level of education. Information, education, and communication about the causes, signs, and nature of mental illnesses would help to reduce stigma.

## Background

Stigma is generally a result of illogical generalization, lack of knowledge, and fear about people who are different from oneself [1]–[3]. Although mental illness is a universal and common health problem [4], communities tend to show stigmatizing behavior towards people with mental illness (PWMI) for one or more of the above mentioned reasons. As a result, PWMI and family members of PWMI find stigma a great challenge to cope with, and international organizations like the United Nations (UN) and the World Health Organization (WHO) strongly suggest that systematic and multifaceted interventions are put into place to fight stigma [5]–[9] against PWMI.

As a consequence of stigma, PWMI usually can have difficulty in maintaining their day-to-day social interactions, which in the worst case may result in them committing suicide [6], [10]–[13]. Stigma is not only a consequence of mental illness but also a factor that interferes with help-seeking behavior, and it may delay treatment-seeking in patients with mental illness [6], [14]–[17] and, as a consequence, the cure and rehabilitation process. For instance, one study conducted in Ethiopia indicated that more than eighty percent of patients with mental illness reported that the community perceives mental illness as a shameful illness, and the same study reported that there was a significant delay in seeking modern treatment for mental illnesses [18].

Mental health is considered a vital element of overall health. The right to mental health care and protection from discrimination is also a human right, but it may be undermined by exclusion of affected individuals through stigma [9], [19]. Although guidelines and conventions on stigma against mental illness are available, much work is required to fight stigma against PWMI. The spectrum of care and the need for rehabilitation services of this particular patient group justifies determined consideration, protection, and advocacy by the respective health care and social systems. In addition, PWMI are disadvantaged with respect to several social determinants of health and exposed to numerous health risks like malnutrition, drug abuse, and homelessness, as well as violence and material deprivation [20]. Moreover, there is a need to fight the negative publicity attached to mental illness in the media and entertainment industries [21]–[23].

Studies from Nigeria, Southern Ghana, and Ethiopia have reported high levels of stigma against PWMI. In these studies, literate participants were more likely to exhibit positive feelings towards the mentally ill than illiterate ones [14], [24], [25]. In contrast, other studies showed that family members with higher levels of education were more likely to report higher levels of stigma [26], [27]. Therefore, education may play negative or positive role for stigma against PWMI or there may be factors which mediate the influence of education on stigma against PWMI. Religion is another important factor with regards to stigma; for example, people of Muslim faith showed less stigma against PWMI than people of other faiths [28]. The difference of stigma against PWMI among different religion followers is because religion usually may dictate some form of explanations of mental illness and may influence the level of stigma a community has against PWMI.

A community's understanding about mental illnesses and its attitude towards PWMI play a paramount role in mental health, because community members act as reinforcing agents for preventive, illness, treatment-seeking, and drug compliance behaviors and also as special rehabilitation agents, because of the chronic nature of mental illnesses. In developing countries like Ethiopia, where mental health services are limited or too scarce and PWMI often delay seeking treatment for their mental illness [29], the community plays an essential role in the treatment and rehabilitation of patients with mental illness. However, community members commonly play a negative role and worsen the consequences of mental illness among patients [30]. Therefore, the aim of this study was to evaluate public stigma against PWMI and the factors associated with stigma in the Gilgel Gibe Field Research Center (GGFRC), which is located in Southwest Ethiopia. The findings of this study will help also organizations working on mental health programs, particularly in fighting stigma against PWMI.

## Methods

### Study design and setting

This community-based, cross-sectional study was conducted at the GGFRC from June to August 2012. The center is located in Southwest Ethiopia, about 50 km from Jimma, on the road from Jimma to Addis Ababa (the capital of Ethiopia), and comprises the area surrounding the Gilgel Gibe Hydroelectric Dam. The center comprises 11 kebeles (the smallest administrative structure in Ethiopia), 3 of which are small towns. In September 2011, the population of the center was 54,538: 15,719 (28.8%) in an urban setting and 38,809 (71.2%) in a rural one [31]. The area serves as a field research center for the Jimma University Health Sciences Research Institute (HSRI).

### Sampling procedure

Of the 11 kebeles, one urban and four rural ones were selected by simple random sampling for inclusion in the study. According to information obtained from the HSRI data center, in June 2012 the five selected GGFRC kebeles comprised a total of 4,268 rural and 1,598 urban households. The proportion of urban and rural households was calculated on the basis of the total number of households in the five kebeles and used to calculate the number of households to be included in each kebele. A simple random sampling technique was used to select the house numbers to be included in the study from the sampling frame obtained at the HSRI data center.

A total of 845 individuals were interviewed in the study community. The maximum sample size was calculated by assuming a 50% level of public stigma–since no data are available about the levels of public stigma in the area–with a 95% confidence interval and considering a tolerable error of 5% and a design effect of 2 as well as adding a 10% non-response rate.

Whenever possible, heads of households were included in the study. Heads of households in this situation were typically spouses (either husband or wife). This might have increased the representativeness of the study since they could have represented their family's thoughts and ideas on the topic. However, individuals aged 18 years and above were included by a lottery method whenever heads of household were absent during data collection.

### Data collection procedure

Data was collected by using an interviewer-administered questionnaire. Training was given to data collectors and supervisors on the contents and procedures of data collection. The training included how to get consent, making familiar to the items of the questionnaire, interviewing techniques, how to administer the questions, principles of confidentiality, and role play of the data collection process. The data was collected by going house-to-house to the randomly selected house numbers.

### Measurement

Public stigma against PWMI was measured with the Community Attitude towards the Mentally Ill (CAMI) scale [32]. The CAMI scale rates a total of 40 items on a 5-point Likert scale (1 = strongly agree to 5 = strongly disagree) and has four subscales, each with 10 items: Authoritarianism (AU), Benevolence (BE), Social Restrictiveness (SR), and Community Mental Health Ideology (CMHI). AU is a ‘view of the mentally ill person as someone who is inferior and requires supervision and coercion.’ BE corresponds to ‘a humanistic and sympathetic view of mentally ill persons’; in this study, a higher BE score corresponded to a less humanistic and less sympathetic (malevolent) view of PWMI. SR means ‘the belief that mentally ill patients are a threat to society and should be avoided.’ Community Mental Health Ideology (CMHI) is ‘the acceptance of mental health services and the integration of mentally ill patients in the community’ [32]; a higher score on the CMHI subscale indicated a rejection of mental health services and the integration of PWMI in the community. Overall stigma against PWMI was computed by summing up the subscales. Negatively stated items were reversely recoded for analysis. Higher scores indicated more stigma against PWMI.

A study conducted in Ghana found good reliability (Cronbach's Alpha) of the CAMI subscales, as follows: BE, α = 0.71; SR, α = 0.73; CMHI, α = 0.75; AU, α = 0.31 [24]. In our study, the reliabilities of the subscales were as follows: AU, α = 0.43; BE, α = 0.50; SR, α = 0.70; CMHI (α = 0.67). When all 40 items were considered, the overall reliability of the CAMI scale was α = 0.79.

A pre-test of the scale was conducted in a similar district outside the study area. The scale was translated and administered in the local languages (Affan Oromo and Amharic) and was back-translated into English to ensure semantic equivalence. In addition to the CAMI scale, demographic and psychosocial characteristics were recorded. Exposure to mental illness information and PWMI was measured by using 9 dichotomous items (for example: message from radio/TV, family/relative with mental illness, ever worked/lived with PWMI, etc) using yes = 1 and no = 0 scores. Higher scores indicated more exposure to mental illness (continuous score). Similarly, a continuous measure of perceived causes (supernatural or psychosocial and biological) and perceived signs of mental illness (example: talking to oneself, suicide attempt, etc) on the basis of yes = 1 and no = 0 were computed by summing up the dichotomous items for each measure.

### Statistical analysis

Each questionnaire was checked for completeness. Data was entered by using EPI-DATA version 3.1 and then exported to STATA version 10.0 for analysis. After data cleaning and editing, the frequency distribution of socio-demographic characteristics was analyzed. Histograms and kernel density plots were used to check the normal distribution of stigma scores. ANOVA (to analyze mean difference among more than two groups) and *t* (to analyze mean difference between two group) tests were also computed to identify the mean difference in public stigma on the basis of socio-demographic and psychographic variables. For each subscales, variables which showed significant statistical association during t tests or ANOVA were included in the multivariate linear regression models. A separate linear regression analysis was performed for each subscale using enter method. A final linear regression model was developed for the overall stigma score. Unadjusted and adjusted standardized regression coefficients were presented for each variable in each model.

A significance level of <0.05 was used to determine a significant association between variables and stigma against PWMI. After the regression analysis, the occurrence of multicollinearity among the independent variables was checked by a variance inflation factor (tolerance) analysis. Then, an interaction analysis was performed to show the multicollinearity effects.

### Ethics statement

Ethical approval was obtained from the Jimma University Research Ethical Review Board. Then, written permission was obtained from the HSRI. Written informed consent was obtained from each study participant. After reading the consent statement by the data collectors, finger prints were obtained from those participants who could not read and write.

## Results

### Socio-demographic characteristics

Of the total 845 study participants, 68.17% were rural residents. Females were over-represented in both the urban (61.71%) and rural subgroups (60.94%). Majority of the respondents were of Muslim faith (71.75% of the urban respondents and 97.05% of the rural ones) and belonged to Oromo ethnic groups (75.09% of the urban respondents and 98.61% of the rural ones).

In general, 76.39% of the rural and 33.46% of the urban respondents were illiterate. Most of the rural respondents were farmers or housewives (94.97%), while in the urban subgroup a higher proportion (52.04%) had other occupations–such as studying or working in small enterprises, as housemaids, or for the government–and only about 48% were farmers or housewives. There were statistically significant differences in the mean age and average monthly family income between urban and rural study participants (P<0.001) (Table 1).

**Table 1:** Socio-demographic characteristics of respondents in the Gilgel Gibe Field Research Center, Southwest Ethiopia, 2012 (N = 845).

Variable	Urban (n_1_ = 269)	Rural (n_2_ = 576)	X^2^, P value or *t* test, P value
	% for n_1_	% for n_2_	
**Sex**			
Female	61.71	60.94	X^2^ = 0.05, P = 0.83
Male	38.29	39.06	
**Marital status**			
Ever been married*	64.68	80.56	X^2^ = 24.97, P<0.001
Never been married	35.32	19.44	
**Religion**			
Muslim	71.75	97.05	X^2^ = 119.85, P<0.001
Others (orthodox, Protestant)	28.25	2.95	
**Ethnicity**			
Oromo	75.09	98.61	X^2^ = 125.40, P<0.001
Others***	24.91	1.39	
**Educational status**			
Illiterate	33.46	76.39	X^2^ = 222.27, P<0.001
Read and write only	7.81	13.02	
Elementary and above	58.74	10.59	
**Occupation**			
Farmer and house wife	47.96	94.97	X^2^ = 253.27, P<0.001
Others**	52.04	5.03	
**Age (mean, SD)**	32.67 (14.16)	39.55 (14.65)	F = 41.27, P<0.001
**Average family monthly income (mean, SD) in ETHB (1 USD = 18.5 ETB)**	545.54 (594.02)	298.56 (204.89)	F = 79.33, P<0.001
**Family size (mean, SD)**	5.01 (2.13)	5.26 (2.18)	F = 2.50, P = 0.11

*Married, divorced, and widowed,

**Private work, student, government employee, house worker (maid),

***Yem, Guraghe, Amhara, Keffa, and Dawro.

### Exposure to and perception of mental illness

The reported lifetime prevalence of mental illness among the respondents was 1.66%, and 9.70% had at least one family member or relative with mental illness either currently or in the past. Among all respondents, 29.23% had been scared by a person with mental illness, and 2.49% reported an experience of physical aggression at some time in their live. In the year preceding the time of the survey, 19.29% of the respondents had heard any type of information about mental illness on the radio; 11.48%, in religious places; and 9.59%, on television. A significant number of respondents (95.15%) had seen a person perceived to have a mental illness, and 14.91% had worked, lived, or studied with a person with mental illness at some time in their live.

The majority of the respondents (75.27%) believed that mental illness can be cured by some means. Among them, 57.08% reported that it can be cured with both traditional and western treatment, while 37.74% believed that it can be cured only with modern treatment. Stress, poverty, and rumination were the most often perceived causes of mental illness, while talking to oneself, self neglect, and talking too much were the most frequently perceived signs of mental illness (Table 2).

**Table 2:** Exposure to mental illness and perceived causes and signs of mental illness in the Gilgel Gibe Field Research Center, Southwest Ethiopia, 2012.

Variables	Number	Percent
**Exposure to mental illness**		
**Ever seen a person with mental illness**	804	95.15
**Ever been scared by a person with mental illness**	247	29.23
**Ever heard about mental illness on radio within the last year**	163	19.29
**Ever worked/lived/studied with a person with mental illnesses**	126	14.91
**Ever heard about mental illness in religious places within the last year**	97	11.48
**Ever had family/relative with mental illness**	82	9.70
**Ever seen information about mental illness on television within the last year**	81	9.59
**Ever been injured by a person with mental illness**	21	2.49
**Ever had a mental illness**	14	1.66
**Belief on cure for mental illness**		
Belief that ‘mental illness can be cured’	636	75.27
Mental illness can be cured only with traditional treatment	33	5.19
Mental illness can be cured only with modern treatment	240	37.74
Mental illness can be cured with both traditional and western healing system	363	57.08
**Perceived causes of mental illness**		
Stress	455	53.85
Poverty	451	53.37
Rumination	356	42.13
God's punishment	177	20.95
Evil spirit	168	19.88
Sinful act	158	18.70
Drug addiction	80	9.47
Physical illness	38	4.50
Germs	9	1.07
**Others (evil eye, failed an exam, and are frightened)**	55	6.51
Perceived signs of mental illness		
Talking to oneself	475	56.21
Self neglect	424	50.18
Talking too much	348	41.18
Strange behaviors	285	33.73
Suicide attempt	192	22.72
Aggression	184	21.78
Restlessness	179	21.18
Sleep disturbance	108	12.78
Unable to learn	33	3.91
Drug addiction	32	3.79
Shivering	24	2.84
Others (calling the evil eye, keeping quiet, to be naked)	39	4.62

### Scores for public stigma against PWMI

The four CAMI subscales (AU, BE, SR, and CMHI) showed statistically significant mean differences in the items setting (urban vs. rural), religion, ethnicity, educational status, and occupation (P<0.001). None of the four subscales showed a significant mean statistical difference between males and females. A significant mean difference was found in the AU and CMHI subscales between the ‘ever been married’ and ‘never been married’ respondents (P<0.05). The overall CAMI score showed statistically significant mean differences in stigma against PWMI in the items marital status (ever been married vs. never been married), setting (urban vs. rural), religion, ethnicity, educational status, and occupation (P<0.01), but again not between males and females. Higher ages and higher scores for perceived supernatural causes of mental illness had a significant positive correlation with stigma against PWMI (P<0.01). On the other hand, higher average family income and higher perceived signs and psychosocial and biological causes of mental illness had a significant negative correlation with stigma against PWMI (P<0.01) (Table 3).

**Table 3:** Stigma mean scores differences based on socio-demographic backgrounds in the Gilgel Gibe Field Research Center, Southwest Ethiopia, 2012.

Variable	1AU			2BE			3SR			4CMHI			Over all stigma		
	M	SD	t/F-test	M	SD	t/F-test	M	SD	t/F-test	M	SD	t/F-test	M	SD	t/F-test
**Sex**															
Female	3.18	0.38	t = 0.02,P = 0.88	2.62	0.44	t = 1.33,P = 0.25	2.43	0.58	t = 0.81,P = 0.37	2.59	0.58	t = 0.02,P = 0.90	2.70	0.35	t = 0.71,p = 0.40
Male	3.18	0.40		2.59	0.41		2.39	0.56		2.58	0.55		2.68	0.32	
**Marital Status**															
Ever married	3.20	0.39	t = 4.94,P = 0.03	2.62	0.43	t = 2.84,P = 0.09	2.42	0.57	t = 0.19,P = 0.66	2.63	0.54	t = 13.56,P<0.001	2.72	0.32	t = 8.29,P<0.01
Never married	3.13	0.38		2.56	0.44		2.40	0.58		2.46	0.62		2.64	0.38	
**Community**															
Rural	3.26	0.40	t = 95.63,P<0.001	2.71	0.45	t = 115.70,P<0.001	2.56	0.61	t = 143.67,P<0.001	2.83	0.50	t = 539.62,P<0.001	2.84	0.30	t = 531.06,P<0.001
Urban	3.00	0.28		2.39	0.27		2.09	0.28		2.07	0.28		2.39	0.18	
**Religion**															
Muslim	3.20	0.39	t = 16.55,P<0.001	2.63	0.43	t = 24.89,P<0.001	2.44	0.58	t = 18.02,P<0.001	2.63	0.56	t = 58.19,P<0.001	2.73	0.33	t = 61.18,P<0.001
Others	3.03	0.31		2.40	0.31		2.17	0.46		2.18	0.43		2.45	0.29	
**Ethnicity**															
Oromo	3.20	0.39	t = 21.53,P<0.001	2.63	0.43	t = 24.07,P<0.001	2.45	0.58	t = 28.14,P<0.001	2.63	0.56	t = 55.57,P<0.001	2.73	0.33	t = 70.02,P<0.001
Others	2.98	0.31		2.38	0.30		2.08	0.37		2.14	0.41		2.40	0.27	
**Educational status**															
Illiterate	3.24	0.38	F = 23.35,P<0.001	2.67	0.45	F = 21.01,P<0.001	2.50	0.60	F = 28.66,P<0.001	2.71	0.55	F = 62.00,P<0.001	2.78	0.33	F = 74.35,P<0.001
Read and write only	3.14	0.43		2.62	0.42		2.48	0.58		2.66	0.53		2.73	0.32	
Elementary and above	3.04	0.34		2.45	0.35		2.17	0.43		2.24	0.47		2.48	0.28	
**Occupation**															
Farmer or housewife	3.22	0.39	t = 35.38,P<0.001	2.65	0.44	t = 40.07,P<0.001	2.48	0.59	t = 42.42,P<0.001	2.68	0.55	t = 109.83,P<0.001	2.76	0.33	t = 124.01,P<0.001
Others	3.02	0.32		2.43	0.33		2.16	0.43		2.20	0.47		2.45	0.28	

1 AU = authoritarianism,

2 BE = benevolence,

3 SR = social restrictiveness,

4 CMHI = community mental health ideology.

### Predictors of public stigma against PWMI

Four independent multivariate models were developed for each of the subscales of the CAMI measures:

#### Authoritarianism

The analysis showed that rural respondents had a significantly higher authoritarianism score than urban participants (std. β = 0.28, P<0.001). Level of education had a significant, inverse statistical relationship with authoritarianism (std. β = −0.15, P<0.01). People who believed that mental illness can be cured had significantly higher authoritarianism scores than their counterparts (std. β = 0.20, P<0.001). As the number of reported signs and symptoms of mental illnesses increased, the tendency to have an authoritarian attitude towards PWMI increased significantly (std. β = 0.16, P<0.001). Respondents who perceived a higher number of psychosocial and biological causes and those who had a higher exposure to PWMI had significantly lower authoritarianism scores (std. β = −0.17, P<0.001, and std. β = −0.18, P<0.001, respectively, for each unit increase of those characteristics).

#### Benevolence

Compared with urban residents, rural residents had significantly higher benevolence scores (i.e. they had a lower humanistic and a less sympathetic approach towards PWMI; std. β = 0.35, P<0.001). When subgroups of respondents were compared that had an educational status differing by one unit, the benevolence score decreased significantly by std. β = −0.12 (P<0.05) units for the subgroup with higher education.

#### Social restrictiveness

Similar to the case for the authoritarianism and benevolence scores, rural residents had also significantly higher (std. β = 0.41, P<0.001) restrictiveness scores, and a higher educational level had a significant, inverse relationship (std. β = −0.12, P<0.05) with social restrictiveness. Individuals with higher number of perceived signs and perceived psychosocial and biological causes of mental illness had significantly lower social restrictiveness scores (P<0.001).

#### Community mental health ideology

Rural residents were significantly more likely to refuse mental health services and to be against integrating PWMI into the community (std. β = 0.59, P<0.001). Significantly lower community mental health ideology scores were obtained among individuals with a belief that mental illness can be cured, those with higher scores for perceived signs of mental illness, and those with higher scores for perceived psychosocial and biological causes (P<0.01).

The multivariate models for authoritarianism, benevolence, social restrictiveness, and community mental health ideology explained 21%, 17%, 23%, and 44% of the variances (adj. R^2^), respectively.

#### Overall stigma against PWMI

For a unit increase in age of respondents, there was a significant decrease in stigma against PWMI by std. β = −0.06 (P<0.05) units. Compared with urban residents, rural residents had a significantly higher stigma score (std. β = 0.61, P<0.001). A significant inverse relationship was observed between the level of education of respondents and stigma (std. β = −0.14, P<0.01), while higher average family income was significantly associated with higher levels of stigma (std. β = 0.07, P<0.05) against PWMI.

Individuals' beliefs that mental illness can be cured in some way was correlated with significantly higher (std. β = 0.07, P<0.01) level of stigma against PWMI. Respondents with higher scores for perceived supernatural causes (std. β = −0.09, P<0.01) and perceived psychosocial and biological causes (std. β = −0.14, P<0.001) had significantly lower stigma levels. Among the predictors of stigma variables, rural residency had the highest coefficient of regression. The regression model for overall stigma explained 44% of the variability (adj. R^2^) (Table 4).

**Table 4:** Predictors of public stigma against PWMI in the Gilgel Gibe Field Research Center, Southwest Ethiopia, 2012.

Variables	Unadjusted β (standardized)	Adjusted β (standardized)
**Age**	0.10**	−0.06*
**Rural community**	0.62***	0.61***
**Educational level**	−0.40***	−0.14**
**Farmer or housewife**	0.36***	−0.01
**Average family monthly income**	−0.15***	0.07*
**Belief that mental illness can be cured**	−0.10**	0.07**
**Perceived signs of mental illness**	−0.12**	−0.03
**Perceived supernatural causes of mental illness**	0.19***	−0.09**
**Perceived psychosocial and biological causes of mental illness**	−0.25***	−0.14***

*P<0.05,

**P<0.01,

***P<0.001.

### Interaction effects

Subsequent analyses found significant interactions between income and education, income and exposure to mental illness, education and exposure to mental illness, and perceived supernatural causes of mental illness and exposure to mental illness. As shown in Figure 1.1, at all three levels of education (low, medium, and high) stigma generally increased as the respondents' income increased, but the increase was statistically significant only at the lower (std. β = 0.28, P<0.001) and medium (std. β = 0.17, P<0.001) levels of education. Similarly, as income increased, stigma against PWMI increased significantly at all three levels of exposure to mental illness information (lower level of exposure: std. β = 0.18, P<0.001; medium level: std. β = 0.13, P<0.01; higher level: std. β = 0.07, P<0.01). The greatest difference in stigma levels between lower and higher income groups was found for those with lower exposure to mental illness information, as shown in Figure 1.2.

**Figure 1:**
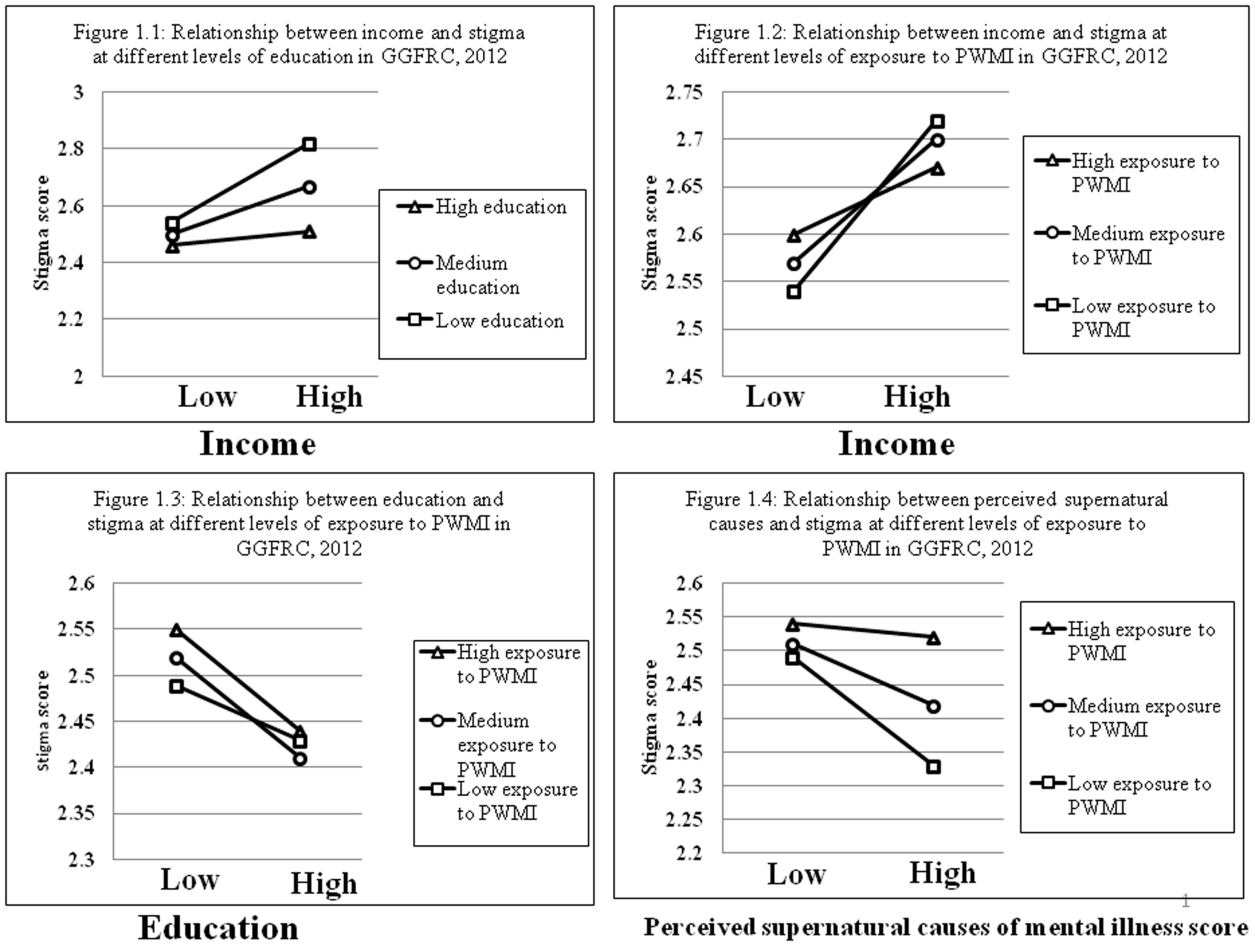
Stigma score at different levels of education and exposure to mental illness with respect to income, education and perceived supernatural causes of mental illness scores in the Gilgel Gibe Field Research Center, Southwest Ethiopia, 2012.

In contrast to the findings regarding income, stigma generally decreased as the educational status increased at different levels of exposure to mental illness information. In particular, there was a statistically significant decrease in stigma at high (std. β = −0.11, P<0.01) and medium (std. β = −0.11, P<0.01) levels of exposure to mental illness information (Figure 1.3). The group with a higher score for perceived supernatural causes of mental illness had significantly lower stigma levels at lower (std. β = −0.16, P<0.001) and medium (std. β = −0.09, P<0.01) levels of exposure to mental illness, as shown in Figure 1.4.

## Discussion

In this study, the strongest predictor of stigma was whether people live in an urban or rural setting: the rural community showed significantly higher levels of stigma against PWMI than people living in an urban area in both the overall score and all four subscales. One explanation for this finding might be that most members of a rural community are illiterate, and another could be a poor dissemination of information on mental illness among rural communities as compared to urban communities. Health service accessibility and availability difference can be also another reason.

One unique finding of this study is that an increase in respondents' level of both perceived supernatural and psychosocial and biological causes of mental illness resulted in a reduction in stigma. This implies that when people have any form of explanation about the causes of mental illness, their stigma level decreases. This is in line with literature reporting that stigma is a result of fear and lack of explanation about an illness and patients [1]–[3], but the way in which supernatural explanations result in lower levels of stigma needs further exploration.

In this study, there was more undermining (higher authoritarianism) but less avoidant (less social restrictiveness) attitudes towards PWMI. The overall level of stigma was lower than in a study in south Ghana [24]. The time differences between the two studies and cultural variability of the study population can be possible factors for the lower level of stigma in the current study. For example, one study has reported being Muslim faith follower was associated with a less stigmatizing attitude towards PWMI [28], although in our study Muslims showed higher stigma scores than non-Muslims. The lower stigma scores among non-Muslims may be caused by the small proportion of non-Muslims in the sample; the difference was not statistically significant in the multivariate analysis.

The mean stigma score was comparable between males and females, i.e., stigma was not associated with gender in either the four subscales or the overall stigma analysis. This implies that there is no need to provide gender-specific anti-stigma interventions in a community. Other studies in Africa and Europe also reported that gender was not a significant factor with regard to stigma against PWMI [24], [33], [34]. A weak negative correlation was found between age and stigma against PWMI; this may be related to the larger sample size in this study.

Education has been found to have negative [26], [27] and positive [14], [24], [25] effects on stigma. In this study, a higher education level was significantly associated with a lower level of stigma. Higher average family monthly income was weakly associated with higher stigma levels. The interaction analysis showed a more synergetic effect of lower education and higher income on stigma level, i.e. respondents with a higher income but lower education level showed higher levels of stigma against PWMI. A potential bias in this finding may be a lower health literacy level in participants with a higher income but lower education, leading to an overestimation of their information level and resulting in inadequate delivery of information by the public or health professionals.

Other studies reported that exposure to PWMI and mental health information reduces stigma against mental illness [35], [36]. In this study, though, there was no significant difference in the overall stigma level between the high exposure and low exposure respondents, the highly exposed subgroup had a significantly lower authoritarianism score against PWMI. A limitation of this measure was its indifference to whether the exposure and experience had been negative or positive.

Besides the authoritarianism subscale, the level of exposure to mental illness information mediated effects on overall stigma among different groups in income, education, and perceived supernatural causes of mental illness. The interaction analysis found that stigma levels increased the most when higher income was accompanied by a lower exposure to mental illness. On the other hand, stigma against PWMI was significantly reduced in respondents with higher exposure to mental illness information and higher education. An explanation for the synergetic effect of these two variables on stigma may be that respondents with higher education are more able to process even complex information and accept new information than others. The level of stigma was also significantly lower among groups with low exposure to mental illness information when the perceived supernatural causes of mental illness score was lower. An explanation could be that respondents with lower exposure were those who received the information from religious places and thus received more sympathetic preaching about PWMI. To understand this effect, studies should be performed to investigate the kind of preaching about mental illness that people hear in religious and traditional healing places.

A significant proportion of respondents believed that mental illness can be cured and this belief was associated with higher scores for authoritarianism but at the same time lower scores for mental health ideology. Believing that mental illness can be cured was positively correlated with a higher overall stigma score against PWMI. This may be due to low levels of understanding of the chronic nature of mental illness and may result in unrealistic expectations that there are fast cures for mental illnesses. Among those respondents who believed that mental illness can be cured, a majority reported that it can be cured with both traditional and western healing systems. This may be helpful for efforts to integrate modern and traditional healing systems in the community. Although it did not have an effect on the overall stigma levels, a higher level of perceived signs of mental illness significantly positively correlated with authoritarianism and negatively correlated with social restrictiveness and community mental health ideology. Other studies also suggested an inverse relationship between the level of understanding about mental illness and stigma [36].

This study has possible limitations. First, some of the stigma items are vulnerable to social desirability bias. Second, the attitudinal object ‘PWMI’ can vary from one person to the other, and the term ‘mental illness’ lacks specificity and is susceptible to different interpretations. Third, the assessment of exposure to mental illness did not specify whether the experience had been positive or negative. Last, average family monthly income was an estimate and not precise.

## Conclusions

More undermining but less avoidant attitudes towards PWMI were found. Stigma against PWMI did not differ between men and women. A higher education level was associated with less stigma against PWMI. Interventions for fighting stigma against PWMI should be targeted more on rural communities. Exposure to mental illness information and a higher education level led to a greater reduction in stigma. Any form of explanation for the cause of mental illness, whether supernatural or psychosocial and biological, reduces stigma against PWMI. The effect of higher expectations that mental illness is a ‘curable illness’ needs further investigation. Interventions also should target people with higher income but a lower level of education. Community mental health information, education, and communication interventions generally are helpful to reduce stigma against PWMI.

## References

[1] Thornicroft G (2006) Shunned: Discrimination against People with Mental Illness Oxford, Oxford University Press, London, England.

[2] Corrigan P (2005) On the Stigma of Mental Illness Washington, D.C., American Psychological Association.

[3] Sartorius N, Schulze H (2005) Reducing the Stigma of Mental Illness. A Report from a Global Programme of the World Psychiatric Association Cambridge, Cambridge University Press.

[4] World Health Organization (2011) Global burden of mental disorders and the need for a comprehensive, coordinated response from health and social sectors at the country level Report by the Secretariat. Geneva, Switzerland.

[5] Canadian mental health association: Stigma and mental illness A Framework for Action by the Canadian Mental Health Association. Available: http://www.cmha.ca/public_policy/stigma-and-mental-illness-a-framework-for-action/. Accessed on 13 September 2012.

[6] Everett B (2006) Stigma: the hidden killer; background paper and literature review. Mood disorders society of Canada, Canada.

[7] Thornicroft G, Brohan E, Kassam A, Lewis-Holmes E (2008) Reducing stigma and discrimination: candidate interventions. International Journal of Mental Health Systems 2: 3. 18405393 10.1186/1752-4458-2-3 PMC2365928

[8] The world health report 2001 (2001) Mental Health: New Understanding, New Hope. WHO, Geneva Switzerland.

[9] United Nations general assembly (1991) The protection of persons with mental illness and the improvement of mental health care. WHO, Geneva Switzerland.

[10] Corry P (2008) Stigma shout: service user and carer experiences of stigma and discrimination. London.

[11] Berzins K (2006) A world to belong to: social networks of people with mental health problems. Public Health and Health Policy, University of Glasgow, Glasgow.

[12] Prior G (2009) Attitudes to Mental Illness 2009 research report. TNS (UK), London.

[13] Prior G (2011) Attitudes to mental illness 2011: research report. TNS (UK), London.

[14] Mohammed K, Zubair I, Isa A, Muktar A (2004) Perception and beliefs about mental illness among adults in Karfi village, northern Nigeria. BMC International Health and Human Rights 4: 3. 15320952 10.1186/1472-698X-4-3 PMC515308

[15] Regier A, Narrow E, Rae S, Manderscheid W, Locke B, et al (1993) The de facto US mental and addictive disorders service system. Epidemiologic Catchment Area prospective 1 year prevalence rates of disorders and services. Archives of General Psychiatry 50: 85–94. 8427558 10.1001/archpsyc.1993.01820140007001

[16] COMPAS survey of Canadians about mental health, mental Illness and depression (1992). Canada.

[17] Rena S (2003) Addressing Stigma: Increasing Public Understanding of Mental Illness. Available: http://knowledgex.camh.net/policy_health/diversity_hr/Documents/addressing_stigma_senatepres03.pdf. Accessed on 21 November 2012.

[18] Eshetu G, Markos T (2011) Patterns of treatment seeking behavior for mental illnesses in south west Ethiopia. BMC Psychiatry 11: 138. 21859455 10.1186/1471-244X-11-138 PMC3170592

[19] United Nations human rights (2010) Monitoring the Convention on the Rights of Persons with Disabilities: Guidance for human rights monitors. UN, New York and Geneva.

[20] Haj-Yahia MM (2002) The impact of wife abuse on marital relations as revealed by the Second Palestinian National Survey on Violence against Women. J Fam Psychol 16: 273–285. 12238410

[21] Beddington J, Cooper CL, Field J, Goswami U, Huppert FA, et al (2008) The mental wealth of nations. Nature 455: 1057–1060. 18948946 10.1038/4551057a

[22] Sartorius N, Schulze H (2006) Reducing the Stigma of Mental Illness: a report from a global programme of the World Psychiatric Association. World Health Organization, Geneva.

[23] Wilson C, Nairn R, Coverdale J, Panapa A (1999) Psychiatry and the media, mental illness depictions in prime-time drama: identifying the discursive resources. Australian and New Zealand Journal of Psychiatry 33: 232–239. 10336221 10.1046/j.1440-1614.1999.00543.x

[24] Barke A, Nyarko S, Klecha D (2011) The stigma of mental illness in Southern Ghana: attitudes of the urban population and patients' views. Soc Psychiatry Psychiatr Epidemiol 46: 1191–1202. 20872212 10.1007/s00127-010-0290-3 PMC3192946

[25] Deribew A, Tamirat Y (2005) How are mental health problems perceived by a community in Agaro town? Ethiop.J.Health Dev. 19: 153–159.

[26] Phelan JC, Bromet EJ, Link BG (1998) Psychiatric illness and family stigma. Schizophr Bull 24: 115–126. 9502550 10.1093/oxfordjournals.schbul.a033304

[27] Oestman M, Kjellin L (2002) Stigma by association: psychological factors in relatives of people with mental illness. Br J Psychiatry 181: 494–498. 12456519 10.1192/bjp.181.6.494

[28] Dols MW (1987) Insanity and its treatment in Islamic society. Med History 31: 1–14. 10.1017/s0025727300046287 PMC1139681 3543559

[29] Bekele YY, Flisher AJ, Alem A, Bahiretebeb Y (2009) Pathways to psychiatric care in Ethiopia. Psychological Medicine 39: 475–483. 18606050 10.1017/S0033291708003929

[30] Corrigan P, Watson A (2002) Understanding the impact of stigma on people with mental illness. World Psychiatry 1: 16–20. 16946807 PMC1489832

[31] Gilgel Gibe Field Research Center. Available: http://www.indepth network.org/Profiles/Gilgel%20HDSS.pdf. Accessed on 24 February 2013.

[32] Taylor SM, Dear MJ (1981) Scaling community attitudes toward the mentally ill. Schizophr Bull 7: 225–240. 7280561 10.1093/schbul/7.2.225

[33] Angermeyer MC, Heiss S, Kirschenhofer S, Ladinser E, Loeffler W, et al (2003) Die deutsche Version des Community-Attitudes-toward-the-Mentally-Ill (CAMI)-Inventars [The German version of the Community-Attitudes-Toward-the-Mentally-Ill (CAMI) inventory]. Psychiatr Prax 30: 202–206. 12768525 10.1055/s-2003-39493

[34] Crabb J, Stewart R, Kokota D, Masson N, Chabunya S, et al (2012) Attitudes towards mental illness in Malawi: a cross-sectional survey. BMC Public Health 12: 541. 22823941 10.1186/1471-2458-12-541 PMC3413535

[35] Corrigan PW, Edwards AB, Green A, Diwan SL, Penn DL (2001) Prejudice, social distance, and familiarity with mental illness. Schizophr Bull 27: 219–25. 11354589 10.1093/oxfordjournals.schbul.a006868

[36] Brockington IF, Hall P, Levings J, Murphy C (1993) The community's tolerance of the mentally ill. Br J Psychiatry 162: 93–99. 8425146 10.1192/bjp.162.1.93

